# Social recommendation model based on user interaction in complex social networks

**DOI:** 10.1371/journal.pone.0218957

**Published:** 2019-07-10

**Authors:** Yakun Li, Jiaomin Liu, Jiadong Ren

**Affiliations:** 1 College of Information Science and Engineering, Yanshan University, Qinhuangdao, Hebei, China; 2 The Key Laboratory for Computer Virtual Technology and System Integration of Hebei Province, Qinhuangdao, Hebei, China; Centre National de la Recherche Scientifique, FRANCE

## Abstract

The user interaction in online social networks can not only reveal the social relationships among users in e-commerce systems, but also imply the social preferences of a target user for recommendation services. However, the current research has rarely explored the impact of social interaction on recommendation performance, especially now that recommender systems face increasing challenges and suffer from poor efficiency due to social data overload. Therefore, applied research on user interaction has become increasingly necessary in the field of social recommendation. In this paper, we develop a novel social recommendation method based on the user interaction in complex social networks, called the SRUI model, to present a basis for improving the efficiency of the recommender systems. Specifically, a weighted social interaction network is first mapped to represent the interactions among social users according to the gathered information about historical user behavior. Thereafter, the complete path set is mined by the complete path mining (CPM) algorithm to find social similar neighbors with tastes similar to those of the target user. Finally, the social similar tendencies of the users on the complete paths are obtained to predict the final ratings of items through the SRUI model. A series of experimental results based on two real public datasets show that our approach performs better than other state-of-the-art methods in terms of recommendation performance.

## Introduction

“With the continuous innovation of Internet technology, new media has ushered in the era of ‘social plus’, which allows various users to communicate with each other and share resources, thus achieving multi-wins”, said Sina weibo chairman of Guowei Cao at the Fifth World Internet Conference held in Wuzhen, China. The Internet has become an open and integrated development platform, and one of its most intuitive manifestations is the commercialization of social media and the socialization of e-commerce [1, 2]. Therefore, under the new situation of complex social networks, determining how to make social recommendations for products has become a hot issue in academic and business research [3].

The existing social recommendation technology mainly utilizes the relationship networks among users or communities to identify the interest preferences of target entities for making recommendations, including content recommendations based on social media [4], neighborhood-based collaborative filtering (CF) [5] and friend recommendations [6,7]. Naturally, in contrast to the traditional research on text content [8], content recommendation processes in social platforms introduce content types that are created and shared by the users themselves, gather the feedback information of other users through the articulated relationships in complex networks, and implicitly infer the user preferences and content popularity [9,10]. Another CF technique has been widely used in social systems [11], and the rationale behind it is to use the rating information of other users in social networks to find the neighbors who have similar tastes to the target user and then recommend items to them. It needs to be emphasized that the CF approach can address some of the limitations of content-based recommendation when the content information of the items is difficult to obtain. Furthermore, friend recommendation refers to helping users find friends of interest in social networks and add them to their contact lists. In these methods, the user social information contained in the complex networks plays a key role in obtaining the user’s real demand to search for suitable products for the user [12].

However, although social networks have been extensively applied in various recommender systems, some of which have achieved good recommendation results to some extent, social overload [13] and network complexity still make it challenging for users to find suitable items, such as goods, music, movies etc. This article breaks through the conventional method of item recommendation [14, 15], and explores the social information that is implicit in the interactions among users in social business systems. Specifically, our paper proposes a novel definition of social interaction and builds an interactive network to describe the relationships among social users in complex networks. Furthermore, the complete path containing the original and terminal users was derived to acquire the social similar neighbors of a target user. Finally, the social rating prediction of potential items was calculated using the defined social distance and social similar tendency.

The main innovations in this article are summarized below.

1. A novel concept of social interaction in complex networks is proposed to construct a weighted social interaction network.
2. The CPM algorithm is proposed to mine the complete paths of a target user and explore user social preferences.
3. The proposed SRUI model is presented in detail to predict the user ratings for unrated items and then make effective social recommendations. Moreover, the recommendation performance is verified based on the experimental results in the Douban and Epinions datasets.
4. Based on the behavioral traces of social users, a new social recommendation model, which is different from the conventional similarity recommendation methods, is designed to improve the accuracy of item recommendations, and also provides diverse support for future research in the field of social recommendations.

The rest of this paper is organized as follows. Section 2 describes the research status of social recommendations and other related work. The related definitions and the framework for SRUI recommendation are presented in Section 3. Section 4 presents the proposed social recommendation model, along with the acquisition of the built social interaction network, the complete path set of the target user, and the final social rating prediction. Experiments on two real datasets are conducted in Section 5. The last section ends the work by summarizing the main contributions of the proposed approach and suggesting future research directions.

## Related work

Many of the current research methods that are most relevant to this study have been proposed for product or message push in social recommendation. For example, Zhang Z et al. [16] proposed a trust and timing model that could make full use of the trust relationships among users and the time series among items to optimize matrix decomposition and improve the quality of social recommendations. The experimental results showed that the above model performs better than the traditional methods, especially for cold users. Similarly, trust enhancement technology, which combines user feedback and trust relationships, was applied by Deng S et al. [17] in service recommendation based on social networks to alleviate the problem of data sparsity in the similarity calculation. Specifically, this approach used matrix factorization to evaluate the degrees of users in social networks, and the random walk algorithm was utilized for the final social recommendation. With more in-depth research on social trust theory, a series of other classic views [18–23]was provided for personalized social recommendations. Additionally, in [24], two novel document-centered methods based on graph partitioning and prototype classification were put forward to sort and select the most relevant social tags from those clusters and classes for social recommender systems. The paper addresses the problem of tag data overload in social media recommendations from the perspective of automatic learning. Chamoso P et al. [25] extracted the relationship information contained in user profiles, job attribute descriptions and user behavior characteristics among users from business- and employment-oriented social networks for job prediction. In order to supply personalized push content for social media sites, Wang G et al. [26] presented a sentiment-aware social media recommendation model that merged the sentiment analysis results of social texts into a collaborative filtering framework, where the inferred sentiment feedback information was classified from the affective texts to improve system performance and increase the recommendation diversity. Nevertheless, these methods still cannot meet the requirements of the rapidly developing recommender systems from the perspective of practical application effects.

In addition, some research on complex social networks is also used in the field of social recommendation. In these studies, the features of the small world [27] and the clustering coefficients [28] in complex networks are exploited to mine the sociality among users and predict the preferences of target users. For instance, Liu G et al. [29] designed an innovative heuristic path mining model based on multiple forecasts that utilized the influence of complex social networks to optimize the user social paths. In the proposed model, the multiple backward local social paths were identified and clustered to generate the final optimal solution with high performance in social service recommendation, and the experimental results indicated that their model was more efficient than the previous algorithms. A novel concept of a hypernetwork with topological features was proposed by Qi Suo et al. [30] to identify users with different roles by analyzing the key attributes of complex social networks. Then, the user rating was re-evaluated based on the similarity of trends in the hypernetwork model, in which users were treated as hyperedges and the products or service as hypernodes. The paper applies the characteristics of a complex network to the field of collaborative recommendation and provides new ideas for social recommendation. Additionally, Fields B et al. [31] adopted complex network technologies to explore the social relevance of musical data for music recommendation and discovery. In addition, although there are some social recommendation models that consider user social information and community structure in social media [32–34], the complex social networks and user interactions in networks are rarely studied in social recommendation methods.

Inspired by the above research status and existing problems, our paper pays special attention to the interaction and social information among users in social recommender systems. Specifically, the behavioral information and interaction relationship of social users are exploited to analyze and mine the preferences of a target user, and then the social similarity tendency is calculated to obtain the rating prediction of items. Based on these analyses, we devise a new approach based on user interaction for social recommendation in complex social networks, aiming to improve the performance of recommendations. Extension experiments based on two real data sets indicate that our method outperformed previous algorithms in terms of accuracy, particularly when the system can provide very few user ratings.

## Definitions and model

### Relevant definitions

Complex networks are suitable for expressing the interactions among social users [35]. Based on the large amount of social data that has grown exponentially [36], this paper defines a complex social interaction network using a directional weighting network. See below for the relevant definitions.

**Definition 1 Social interaction** is considered to be a social interactive relationship among users in a recommender system, such as, comments, forwards, push messages, blog posts, other social services etc. Thus, interaction represents the links among users in complex networks. Besides, the direction of social interaction is that the initiator of social behavior points to the recipient. For example, if user A comments on Weibo contents posted by user B, the direction of social interaction is from A to B.

**Definition 2 Social interaction network(SIN)** is a directed relationship network built up through the social interaction among the users in complex networks. Generally, the nodes in a network represent the social users, the edges represent the interactions among the users, and the weights are expressed as the user ratings of interactions. The definition of an SIN is shown below.

SIN={NODES,EDGES,WEIGHTS} (1)

The SIN can be represented as a triple, where NODES is the sequence collection of the users in a complex social network denoted by U = {u_1_, u_2_, ⋯, u_m_}, EDGES is the sequence collection of the interaction relationships denoted by $I=\{i_{u_{1},u_{2}},i_{u_{3},u_{4}},\cdots ,i_{u_{k},u_{k+1}}\}(u_{k}\in U)$, and WEIGHTS is the rating sequence set denoted by $R=\{r_{u_{1},u_{2}},r_{u_{3},u_{4}},\cdots ,r_{u_{k},u_{k+1}}\}(u_{k}\in U)$. What needs to be explained is that the interaction $i_{u_{k},u_{k+1}}$ is not equal to the interaction $i_{u_{k+1},u_{k}}$ in our paper. That is, the user u_k_ makes an interaction with the user u_k+1_ called $i_{u_{k},u_{k+1}}$, and the user u_k+1_ also makes an interaction with the user u_k_ called $i_{u_{k+1},u_{k}}$; therefore, the $i_{u_{k},u_{k+1}}$ and $i_{u_{k+1},u_{k}}$ are two differentinteractions.

**Fig 1:**
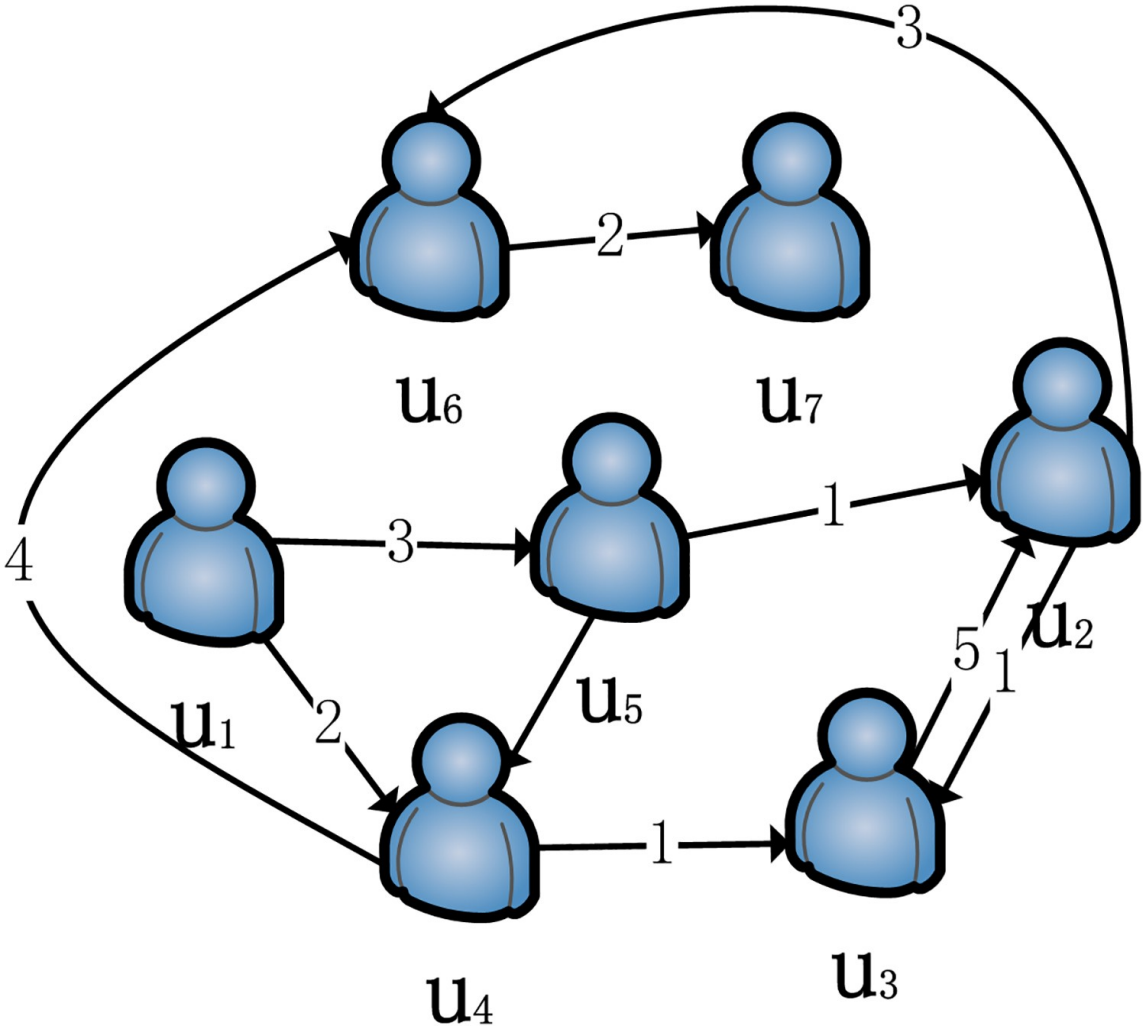
A simple social interaction network.

**Definition 3 The reachability** symbolized by→is the existence of a directional path that can be connected from one user to another in an SIN. For example, in a simple social interaction network, as shown in Fig 1, u_1_ → u_5_ and u_4_ → u_7_ are both reachable.

**Definition 4 Original users** (**u**_**o**_) are those users who have no other user social interaction with them or those whose in-degree is zero in the SIN. For example, in Fig 1, u_1_ is an original user.

**Definition 5 Terminal users** (**u**_**T**_) are those who have not made social interactions with any other users or those whose out-degree is zero in the SIN. As shown in Fig 1, u_7_ is a terminal user.

**Definition 6 The complete path (CP)** refers to a directed sequence path containing the target user in the SIN, where the first user is the original user and the last user is the terminal user and each user can only appear once. In this case, the target user u belongs to U.

$${cp}_{k}(u)=\{u_{O},\cdots u,\cdots u_{T}\}$$ (2)

For example, in Fig 1, for the user u_5_, cp_1_(u_5_) = {u_1_, u_5_, u_4_, u_6_, u_7_}, cp_2_(u_5_) = {u_1_, u_5_, u_2_, u_6_, u_7_} and cp_3_(u_5_) = {u_1_, u_5_, u_4_, u_3_, u_2_, u_6_, u_7_} are all complete paths. In addition, two different users can have the same complete path.

**Definition 7 Complete path set** refers to the collection of all complete paths of the target user in the SIN. The specific definition is as follows. N is the number of all the complete paths of the target user u.

$$CP(u)={⋃}_{k=1}^{N}{cp}_{k}(u)=\{{cp}_{1}(u),{cp}_{2}(u),\cdots ,{cp}_{N}(u)\}$$ (3)

**Definition 8 Social similar neighbors** are users in the complete path set of the target user.

**Definition 9 Social distance (SD)** is the distance between any two reachable users and includes the number of interactive edges on some complete path. K is the kth complete path of the target user u (k ∈ Z^+^). |i_u,v_| is the number of interactive edges between users u and v on the complete path. If users u and v are adjacent users on some complete path, then the value of |i_u,v_| is 1.

$${sd}_{u,v}=|i_{u,v}|(v\in {cp}_{k}(u))$$ (4)

**Definition 10 Social similar tendency (SST)** refers to the degree of social similarity of a user to the target user in some complete path. It expresses the similar preferences between another user and the target user through user interactions. The SST value is proportional to the weight ratings of the interactive edges and inversely proportional to the social distance between two users.

$${SST}_{u,v}=\frac{1}{k}∙\sum_{CP(u)}\frac{{\tilde{r}}_{u,v}}{1+{sd}_{u,v}}$$ (5)

In the above definition, sd_u,v_ is the social distance between user u and user v, k is the number of complete paths containing user u and user v, and ${\tilde{r}}_{u,v}$ is the sum of weight ratings for the interactive edges between user u and user v on the complete path. It is defined as follows.

$${\tilde{r}}_{u,v}=\sum_{{cp}_{k}(u,v)}r_{u_{m},v_{n}}(u_{m},v_{n}\in {cp}_{k}(u))$$ (6)

After obtaining the social similar tendency between two users, this paper can predict the items or products for the target user in the model. The social rating prediction is the predicted rating value of theuser items or products and is obtained by using the mean of the social similar tendency and weight ratings.

$${SRP}_{u,v}={SST}_{u,v}*|{SST}_{u,v}-{\overset{-}{r}}_{u,v}|$$ (7)

Specifically, in the formula, ${\overset{-}{r}}_{u,v}$ means the average of all the weight ratings for the interactive edges between user u and user v on the complete path of the target user. With the social rating predictions of all the items, inspired by the user-based recommendation method [37], our paper sorts the predictions through the Top-N algorithm [38] and then recommends the corresponding items to the target user.

For example, in Fig 1, we assume that u_4_ is the target user and there are two complete paths, cp_1_(u_4_) = {u_1_, u_4_, u_6_, u_7_} and cp_2_(u_4_) = {u_1_, u_4_, u_3_, u_2_, u_6_, u_7_} respectively. These paths form the complete path set for the target user u_4_, known as CP(u_4_) = {cp_1_(u_4_), cp_2_(u_4_)}.

Next, we use the previous definition in formulas (4) and (6) to calculate the social distance ${sd}_{u_{4},v}$ and the sum of the weight rating ${\tilde{r}}_{u_{4},v}$ between the target user u_4_ and other users v(u_1_, u_2_, u_3_, u_6_, u_7_) on each complete path, as shown in the second and third columns of Table 1.

**Table 1:** The recommendation instance of target user u_4_.

	${\left({sd}_{u_{4},v},{\tilde{r}}_{u_{4},v}\right)}_{{cp}_{1}(u_{4})}$	${\left({sd}_{u_{4},v},{\tilde{r}}_{u_{4},v}\right)}_{{cp}_{2}(u_{4})}$	${SST}_{u_{4},v}$	${\overset{-}{r}}_{u_{4},v}$	${SRP}_{u_{4},v}$
u_1_	(1,2)	(1,2)	1	2	1
u_2_	—	(2,6)	2	3	2
u_3_	—	(1,1)	0.5	1	0.25
u_6_	(1,4)	(3,9)	2.125	3.25	2.391
u_7_	(2,6)	(4,11)	2.1	2.833	1.539

Finally, ${SST}_{u_{4},v},{\overset{-}{r}}_{u_{4},v}$ and the final social rating prediction ${SRP}_{u_{4},v}$ are calculated separately based on what has been obtained and formulas (5) and (7). The detailed results are shown in Table 1 below.

### Framework of SRUI recommendation

**Fig 2:**
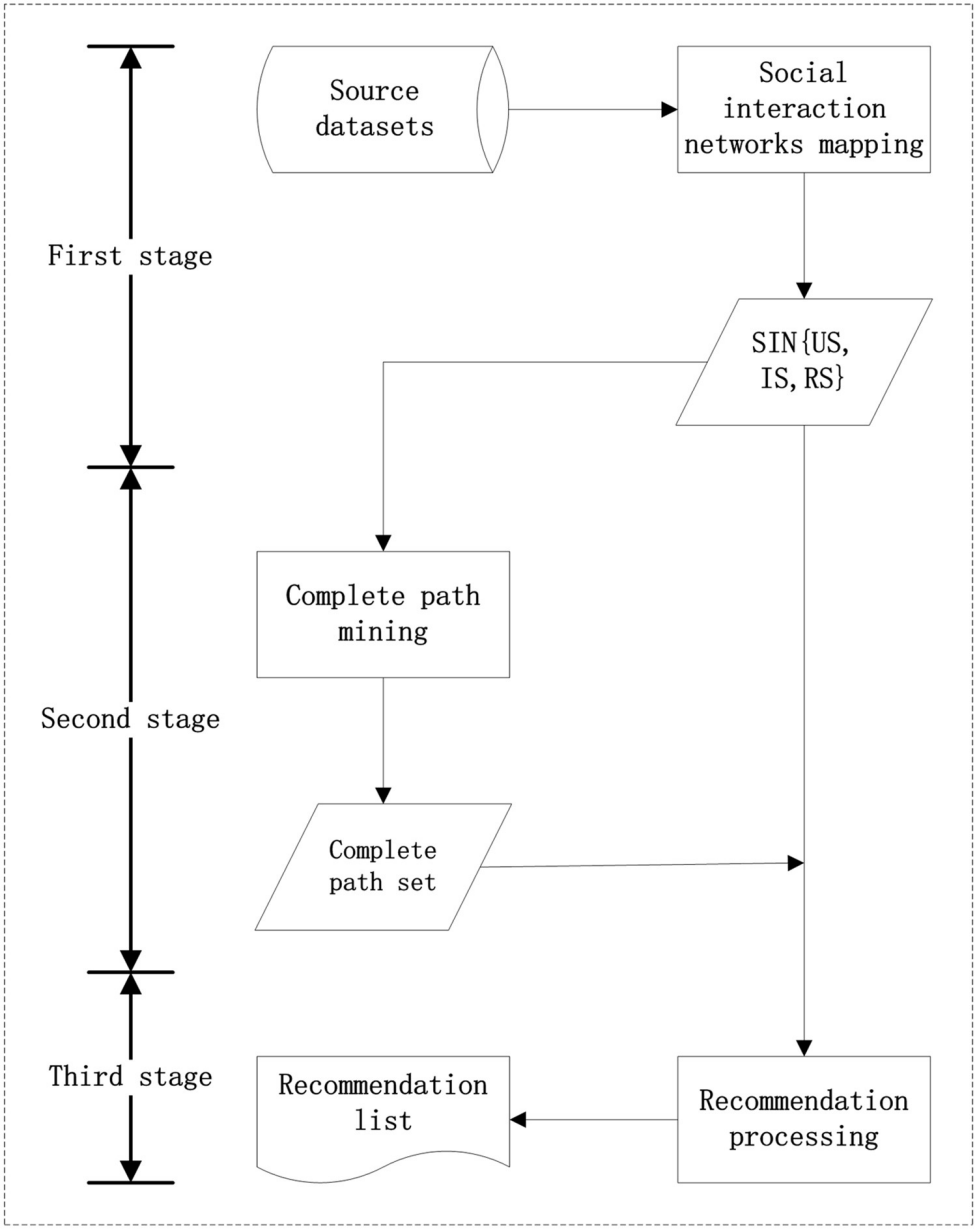
Framework of SRUI recommendation.

The framework of SRUI recommendation is mainly completed in three stages. These stages are collaborative to analyze the user interaction in social media and obtain the social preferences of the target user so as to make effective item recommendations. The framework description of the SRUI model is presented in Fig 2. In the initial stage, we preprocess the source datasets to structure the interaction information. Based on the social interaction network mapping algorithm, the interaction information among social users can be mapped into different sequence sets. Then, through analyzing and leveraging the social interaction networks, this article explores the complete paths of the target user to further learn about his potential social preferences. Finally through recommendation processing, the proposed SRUI algorithm calculates the social similar tendency and social rating prediction of the target user to generate the recommendation result.

In summary, the main contribution of the proposed framework is to model the user interaction network by analyzing and utilizing the social user’s behavioral records (comments, retweets, sharing, etc.) in the recommendation system, and to define some novel networking concepts such as social interaction, complete path, etc. Based on the proposed SRUI model, highly rated items are selected to form the final recommendation lists. Moreover, our approach realizes significant improvements in term of accuracy and efficiency in complex networks, and also enriches the diversity of research on the application of recommendation systems.

## Proposed social recommendation model

Traditional social recommendation methods mostly use user trust networks to push information to target groups timely and accurately [39]. Most of these methods are based on the collaborative filtering algorithms or their variations, requiring the systems to provide sufficient rating information. However, in the complex environment of social networks, many users regard their personal information as private, and it is difficult for recommender systems to obtain user trust networks and interest preferences, which leads directly to a dramatic reduction in recommendation efficiency [40]. Therefore, this article proposes a novel social recommendation method that builds a new user relationship network by leveraging the interaction relationships among social users in complex networks. Moreover, this method acquires users’ preferences to the maximum extent to draw on all of the useful social information in the social network and obtain a better estimate of the real preferences of the target user. The proposed model breaks through the inherent bottleneck in the traditional recommender systems, and establishes a novel networking model to provide recommendations. The experiments in the next section also verify that our algorithm alleviates the data sparsity problem to some extent and improves the recommendation accuracy. The interactive networks are mapped through a depth-first search for the adjacent nodes of the original user. The detailed pseudocode of the social interaction network establishment (SINE) algorithm is shown below.

**Algorithm 1**: **The social interaction network establishment (SINE) algorithm**

**Input**: Original social interaction datasets D.

**Output**: SIN(US, IS, RS): user sequence US; interaction sequence IS; rating sequence RS.

**Process**:

(1) **Initial** User sequence US = {u_o_}; Interaction sequence IS = ∅;

 Rating sequence RS = ∅   // u_o_ is any original user in the datasets

(2) **For** each element e_k_ ∈ D, do

(3)  **While** (isUserNode(e_k_) ⩵ true)

(4)   **If** (e_k_.isNextAdjacentUser(u_n_))   //u_n_ ∈ US

(5)    **For** ($the\;interaction\;i_{u_{n},e_{k}}!=\emptyset$)

(6)     $i_{u_{n},e_{k}}.addToSequence(IS);r_{u_{n},e_{k}}.addToSequence(RS)$

(7)    **End for**

(8)   e_k_.addToSequence(US); k++

(9) **End for**

In the first line of the algorithm three sequence sets are initialized, namely the user sequence, interaction sequence and rating sequence, where the user sequence is initialized to any original user. In lines 2 to 9, the mapping process is based on the initialized original user u_o_, and deeply traverses the data sets D to build the user interactive network. Specifically, if the data element e_k_ in the data sets is nonempty, lines 2 to 4 of the algorithm will determine whether it is an adjacent user to one of the users in the set of user sequence US. As shown in lines 5–7, if there is an interaction between the two users, the edge and weight in the social interaction network are separately constructed and added to the corresponding sequence set. Finally, the adjacent user is added to the user sequence set, and the next loop continues to map the network.

To locate the target user’s preference information, we analyze their social situation across the interactive network. Mining the complete path of the target user in an interactive network, which provides a lot of implicit feedback about the user’s personalized social characteristics, has become one of the key steps in social recommendation. Therefore, this paper proposes a CPM algorithm to excavate the complete path of the target user. In detail, the CPM algorithm is mainly composed of three parts. First, the method traverses the interactive network to obtain the original and terminal user sequence sets. Then, the users on the path are stored in the user list by marking a directed path from the original user to the terminal user. Finally, if the target user is in this user list, then this path is the complete path. Otherwise, the algorithm starts searching again. The specific process of the CPM algorithm is described as follows.

**Algorithm 2**: **CPM algorithm**

**Input**: SIN(US, IS, RS); The target user u.

**Output**: Complete path set of the target user uCP(u).

**Process**:

(1) **Initial** UserList UL; Original user set U_O_ = ∅; Terminal user set U_T_ = ∅; cp_n_(u, v) = ∅

(2) **For** (each u_k_ in US)

(3)  **If** (Indegre(u_k_) ⩵ 0)

(4)   u_k_.addUser(U_O_)

(5)  **If** (Outdegre(u_k_) ⩵ 0)

(6)   u_k_.addUser(U_T_)

(7) **End for**

(8) **For** (each original user u_o_ ∈ U_O_)

(9)  u_o_.insertList (UL);

(10)   **For** (each u_k_ not in U_O_)

(11)   u_o_.depthTraverse(SIN)

(12)   **If** u_k_.isNextAdjacentUser(u_o_))

(13)    u_k_.insertList (UL))

(14)   **If** (u_k_ in U_T_)

(15)    u_k_.insertList (UL)); break

(16)   **End for**

(17)  **If** (u isExsitIn(UL))

(18) cp_n_(u) = UL.traverseList()

(19)  Form(CP(u))

(20) **End for**

The relevant variables are initialized by the algorithm in the first line. In lines 2 to 7, according to the in-degree and out-degree of the user nodes in the interactive network, the algorithm saves the original and terminal users to the corresponding U_O_ and U_T_ sets respectively. For one original user in U_O_, lines 8–9 insert the user into the user list UL. From lines 10 to 16, the algorithm deeply traverses the interactive network from the original user to the terminal user, and then inserts the users on the path into the UL. Line 17 determines whether the given target user is in the list UL, and the complete path is obtained in line 18. Finally, lines 19–20 form the complete path set for the target user u, and end the algorithm.

During the entire process described above, the social interaction network and the complete path set of the target user are obtained. Then, on the basis of the previous definitions and analyses, we propose a novel SRUI method to obtain the rating prediction value for the recommendation, of which the algorithm description is presented as follows.

**Algorithm 3**: **The SRUI algorithm**

**Input**: SIN(US, IS, RS); target user u_t_

**Output**: The social rating prediction SRP

**Process**:

(1) **Initial** CP(u_t_) = {∅}; Array temp_sd; Array $temp\_\tilde{r}$

(2) **For** target user u_t_ ∈ US

(3)  **CPM**(SIN, u_t_)   //CP(u_t_) is obtained

(4)   **For** (int j=1; j<=CP(u_t_).Count; j++)  //j is the jth complete path cp_j_(u_t_)

(5)    **Array** $temp\_sd[j]=|i_{u_{t},u_{k}}|$  // u_k_ is the user on complete path cp_j_(u_t_)

(6)    **Array**$temp\_\tilde{r}[j]$.sumRate(u_t_, u_k_)

(7)   **End for**

(8)  **For** any user u_p_ ∈ cp_n_(u_t_)

(9)   ${SST}_{u_{t},u_{p}}=calculate\_SST(temp\_sd[j],temp\_\tilde{r}[j])$ // social similar tendency is obtained

(10)   ${SRP}_{u_{t},u_{p}}=compute\_IRP({SST}_{u_{t},u_{p}},{\overset{-}{r}}_{u_{t},u_{p}})$  // the item rating prediction

(11)  **End for**

(12) **End for**

In line 1, the algorithm initializes the complete path set CP(u_t_), social distance array temp_sd and weight ratings array $temp\_\tilde{r}$. Lines 2–7 of the algorithm acquire the social distance and weight ratings between the target user u_t_ and any user u_k_ on the complete path, wherein line 3 calls the CPM algorithm to obtain their complete path set CP(u_t_). In the end, the social similar tendency and item rating prediction are calculated successively according to formulas (5) and (7) in lines 8–12.

As we can see from the above algorithms that differ from the conventional similarity recommendation methods, the fundamental principle of the proposed model can make full use of the user’s comments, retweets and other online behavioral information in complex networks to map the target user’s social interactive network. Then, based on this information, our method can gather more similar neighbors by mining the complete paths of the target user to better model and locate the user’s interests and preferences and improve the prediction accuracy of the products. Obviously, according to the previous analysis, each process of the social recommendation model is a significant part of the recommendation framework, especially the SRUI algorithm, which plays a key role.

## Experiments and analysis

In this section, a series of experiments was conducted to verify the effectiveness of the proposed method with two real datasets. Then, we adopted two commonly used evaluation metrics to compare the performance of the recommendation in our approach with that of other advanced methods. The experiments were performed on a computer with an Intel(R) Core(TM) i5-4590 CPU @3.30GHz and 4.00GB of RAM operating under the 32 bit Windows 10 Professional operating system, and the algorithm was implemented in the Java language and the Eclipse programming environment.

### Experimental datasets

In accordance with the method proposed in this paper, we use the Douban and Epinions datasets as our experimental data sets. Both datasets were generated in social networking environments. The data sets were preprocessed to remove some unnecessary and useless information. The description of the datasets is introduced in detail below.

Douban is a Chinese social service website that integrates a taste system, expression system and communication system and is dedicated to discovering useful things in people’s lives. In the Douban dataset, all the content, classification, filtering and sorting tasks are generated and determined by social users. You can freely issue and search for comments and recommendations for items in this system. The open source Douban dataset [41] was collected by the LibRec team in the java-based cross-platform recommendation tool library and specifically contains 16,830,839 ratings for 58,541 items by 129,490 users.

Epinions is a social review site where users are free to comment on items by specifying a positive integer rating in the range of 1–5. The publicly available Epinions dataset [42]was collected by P. Massa and P. Avesani. The dataset we employed consisted of 49,000 users who generated 664,000 ratings of 139,000 different items and some other interactive information.

### Baseline methods and evaluation metrics

In order to evaluate the effectiveness of the proposed method and the recommendation results, several state-of-the-art algorithms were compared with our approach, including user-based collaborative filtering (UCF) [43], social network-based recommendation (SNR) [17], multi-view user preference learning (MVUPL) [44], joint social and content recommendation (JSCR) [45] and graph-regularized matrix completion (GRMC) [46]. Specifically, the basic principle of UCF recommendation is to first find the neighbor set similar to the target user’s tastes and preferences according to all the users’ rating information on items. Then, based on the historical preference information of K neighbor users, a recommendation is made for the target user. Another idea of the comparative SNR method described above is to use matrix decomposition to measure the trust value among the users in the social network, and generate the final recommendation by the random walk algorithm. The MVUPL method has provided a multi-view user preference learning mechanism that takes advantage of user social relationships, rating information, item side information and tagging information to present social recommendations. In addition, a JSCR framework is designed to construct a user-content matrix and fill in the cold user-video entries to provide the foundation for a recommendation in an online social network. The GRMC algorithm utilizes user social networks and graph-regularized matrix completion to infer the user models and thus solves the optimization problem to improve the performance of recommender systems.

For the sake of evaluating the prediction accuracy of all the algorithms in the experiment, the dataset is divided into a training set and a testing set. Then, the root mean square error (RMSE) and mean absolute error (MAE) are adopted to measure the accuracy of the rating prediction in the recommender system. These two evaluation metrics are currently the most popular in the rating prediction field. The RMSE between the predicted rating and the true rating is defined by the following formula.

$$RMSE=\sqrt{\frac{\sum_{u,v\in \varphi }{{(SRP}_{u,v}-r_{u,v})}^{2}}{\left|\varphi \right|}}$$ (8)

Where |φ| is the number of predicted ratings in the test set φ, r_u,v_ represents the true rating of item i_u,v_, and SRP_u,v_ denotes the predicted rating of item i_u,v_. The MAE is another commonly used evaluation metric, as shown below.

$$MAE=\frac{1}{\left|\varphi \right|}\sum_{u,v\in \varphi }\left|{SRP}_{u,v}-r_{u,v}\right|$$ (9)

Compared to the MAE, the RMSE can punish large errors in different proportions, but they both reflect the prediction accuracy of items in different ways; obviously, the higher the values are, the lower the performance of the recommendation system. Therefore, in our experiment, we can use both the MAE and RMSE to evaluate the recommendation results.

### Experimental results and analysis

Based on the above analysis of social interaction, contrast experiments are conducted to compare the performance of these six methods based on two datasets. Moreover, in order to obtain the experimental results more accurately, the number of social similar neighbors (SSN) is set from small to large as 5, 15, 25, 35, 45, 55, 65, 75, 85 and 95. In addition, the experiments were carried out under the same environment, and the specific results and analyses are as follows.

**Fig 3:**
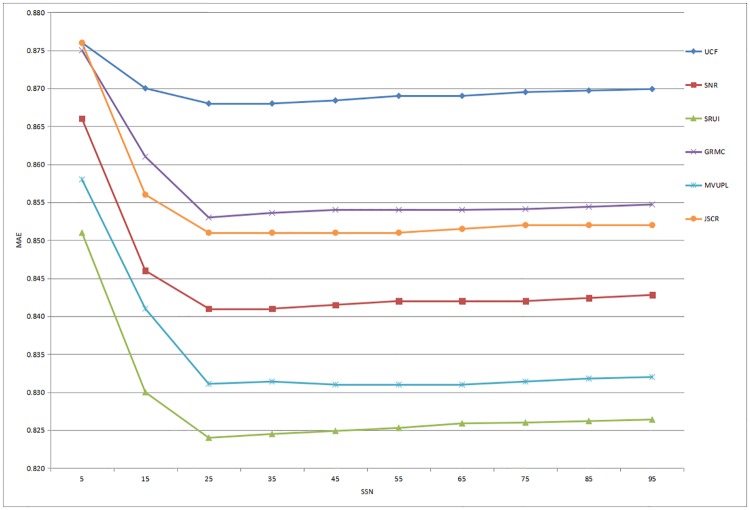
Prediction accuracy of all the comparison methods for different numbers of social similar neighbors based on the Douban dataset (MAE).

**Fig 4:**
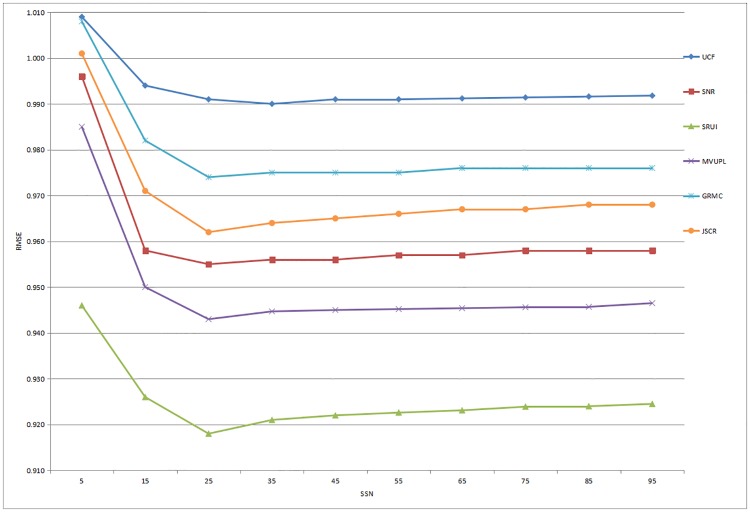
Prediction accuracy of all the comparison methods for different numbers of social similar neighbors based on the Douban dataset (RMSE).

The recommendation performance of the six methods based on the Douban dataset is illustrated in Figs 3 and 4. The results show that as the number of SSN increases, the values of the MAE and RMSE gradually decrease and the rating prediction becomes closer to the real value. When the SSN reaches approximately 25, the performance of the recommender system reaches a maximum and then steadily stabilizes. Furthermore, the UCF method consistently performs the worst in our comparative experiments, and the proposed method performs significantly better than other advanced approaches based on the prediction accuracy.

**Fig 5:**
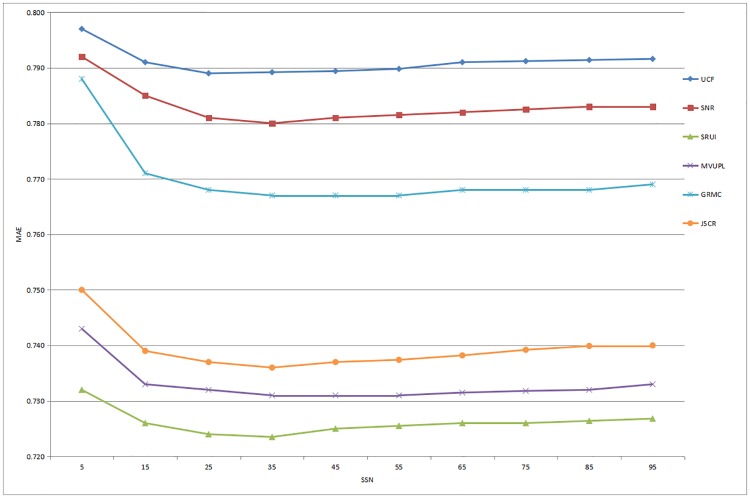
Prediction accuracy of all the comparison methods for different numbers of social similar neighbors based on the Epinions dataset (MAE).

**Fig 6:**
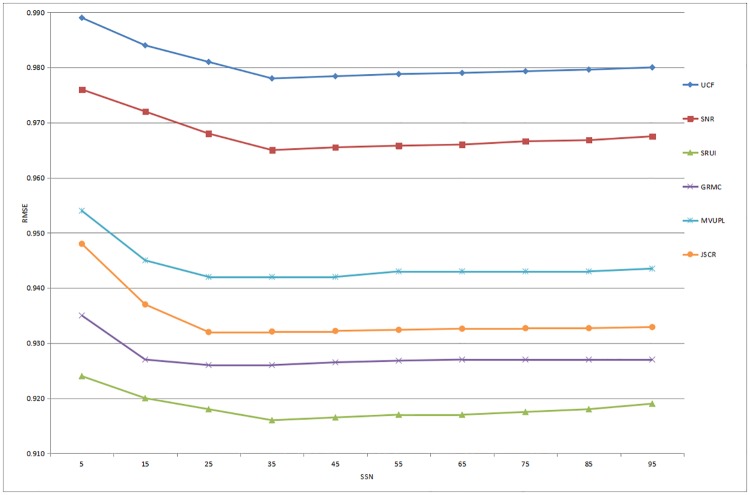
Prediction accuracy of all the comparison methods for different numbers of social similar neighbors based on the Epinions dataset (RMSE).

Figs 5 and 6 show the comparative results for the MAE and RMSE of the six methods based on the Epinions dataset. From Figs 5 and 6, we can find that the recommendation performance of three methods is relatively stable and that the SRUI and JSCR perform better than most other social recommendation approaches with increasing number of social similar neighbors. When the number of SSN is approximately 35, the accuracy of the rating prediction for all six methods reaches the highest value. Moreover, the analysis based on the above Figs 5 and 6 suggests that the proposed method maintains the best performance throughout the experiment.

In summary, these experimental results indicate that compared with the rating predictions of existing methods, the recommendation results obtained through the proposed social recommendation model based on user interaction are more accurate for both datasets, especially when the number of SSN is between 25 and 35. Therefore, our approach is proved to be an effective recommendation service that meets the personalized needs of users by utilizing and analyzing the user interaction information in complex social networks.

## Conclusion and future work

In many recommender system methods, the similarity calculation is frequently used. In contrast to most of the current similarity recommendation methods, a novel social recommendation model based on user interaction is proposed to improve the accuracy of the social recommender system in this article. The main innovation of our model is obtaining the user social similar tendency by proposing the new concept of social interaction and an interaction network model in which user social preferences are learned by grouping similar users along the complete paths of the target user in complex social networks. Specifically, the interactive network is first mapped to define and express the social interaction among users, and the SINE algorithm is exploited to obtain the user, rating and interaction sequence sets. Second, the CPM method is presented to search and generate a complete path set for the target user, which can provide and find social neighbors with similar tastes. Finally, the proposed social recommendation model makes predictions for the item ratings. In addition, along with the experiments, an empirical evaluation of our approach was conducted to compare to several state-of-the-art methods using the Douban and Epinions datasets. The results indicate that the performance of the SRUI algorithm is superior to that of the previous methods. However, the social recommendations based on user interaction may change significantly in different time periods. Therefore, in the near future, we will focus on the dynamic learning of user behavior that changes over time.
